# Impact of weekly frequency of high‐intensity interval training on cardiorespiratory, metabolic, and performance measures in recreational runners – An exploratory study

**DOI:** 10.14814/phy2.70573

**Published:** 2025-09-21

**Authors:** Mascha Lenk, Manuel Matzka, Lukas Lauber, Philipp Kunz, Billy Sperlich

**Affiliations:** ^1^ Integrative and Experimental Exercise Science and Training, Department of Sport Science University of Würzburg Würzburg Germany

**Keywords:** cardiorespiratory fitness, high‐intensity interval training, physiological adaptations, recreational exercisers, running economy, submaximal heart rate, time‐to‐exhaustion, training frequency, VO_2max_

## Abstract

This exploratory study examined the effects of varying frequencies of 4 × 4‐min high‐intensity interval training (HIIT) sessions (once, twice, or thrice weekly) on cardiorespiratory, metabolic, perceptual, and performance variables in recreationally active individuals. Twenty‐six participants (VO_2max_: 51.3 ± 7.1 mL·kg^−1^·min^−1^) completed a six‐week intervention, utilizing individualized HIIT protocols based on maximal treadmill speed and heart rate. Pre‐ and post‐assessments involved an incremental treadmill test evaluating maximal oxygen uptake (VO_2max_), time to exhaustion (TTE), ventilatory threshold (VT_1_), submaximal heart rate, and running economy (i.e., submaximum oxygen uptake). Adherence rates ranged from 91% to 100%. Significant improvements in VO_2max_ and TTE were observed across all training frequencies (*p* < 0.05), with moderate‐to‐large effect sizes (Cohen's d [95% CI] for VO_2max_ = 0.09–0.61 [−0.56 to 1.42]; TTE: 0.02–0.77 [−0.65 to 1.61]). The largest effects (*d* > 0.5) were found for VO_2max_ and TTE in the groups training two or three times per week. 2–3 weekly sessions of 4 × 4‐min HIIT may be effective for improving VO_2max_ and TTE in recreationally active individuals, with no clear additional benefit from increasing the frequency to three sessions per week. These preliminary findings highlight the potential of twice‐weekly HIIT as a practical and time‐efficient training strategy, while acknowledging limited precision.

## 1 INTRODUCTION

High‐Intensity Interval Training (HIIT) is broadly acknowledged for enhancing cardiovascular, metabolic, and muscular functions, making it both effective and time‐efficient for a wide range of populations, including athletes, recreational exercisers, and sedentary individuals (Duking et al., 2020; Engel et al., 2018; Helgerud et al., 2007; Messler et al., 2018; Reuter et al., 2023; Zinner et al., 2018). HIIT encompasses a variety of formats, from repeated sprint interval training (SIT) to longer intervals (e.g., 3 × 8 min (Seiler et al., 2013)). Among these formats, the 4 × 4‐min protocol is particularly well studied due to its demonstrated benefits in improving cardiovascular fitness and its adaptability across diverse groups, such as elite athletes (Stoggl & Sperlich, 2014) or recreational exercisers (Zinner et al., 2018). This protocol typically involves 4 min of high‐intensity exercise at 90%–95% of maximal heart rate, interspersed with 3 min of active recovery, repeated four times (Helgerud et al., 2007).

Despite the well‐established benefits of various forms of HIIT, the optimal frequency of training sessions—an essential component in the training process—has received comparatively less scientific attention. Altering the frequency of HIIT sessions can significantly influence the overall training outcomes, impacting factors such as recovery, adaptation, and performance improvements (Dalleck et al., 2010; Kavaliauskas et al., 2017; Stavrinou et al., 2018). Indeed, several studies have demonstrated a lack of enhancement in cardiorespiratory adaptation, specifically in terms of maximum oxygen uptake (VO_2max_), following interval training regimens performed once or twice weekly (Dalleck et al., 2010; Kavaliauskas et al., 2017). Additionally, increasing session frequency from twice to thrice weekly has been shown to improve cardiorespiratory fitness, body fat reduction, and lipid metabolism (World Medical Association, 2013).

Its time‐efficient nature makes HIIT particularly appealing for recreational exercisers seeking health and fitness benefits, though time constraints often limit many adults (Dalleck et al., 2010) to just one session per week. Despite the popularity of HIIT, there is limited data on how different frequencies of HIIT (once, twice, or thrice weekly) affect key endurance variables such as VO_2max_, ventilatory threshold, running economy (i.e., submaximal oxygen uptake), and time‐to‐exhaustion. The present study addresses this gap by evaluating the effects of one, two, or three weekly 4 × 4‐min HIIT sessions over 6 weeks on these variables, providing insights into the optimal training frequency for recreationally active individuals.

## 2 METHODS

### 2.1 Participants

26 participants (14 female; age: 23 ± 3 years; VO_2max_: 51.3 ± 7.1 mL·kg^−1^·min^−1^; Body Mass Index: 24.0 ± 2.3 kg·m^−2^) out of 44 initially recruited recreationally active individuals completed the intervention and testing procedures (59.1%). The remaining participants withdrew due to time constraints or illness such as colds or flu. Participants were not sedentary but engaged in regular, structured physical activity and were considered highly active on a recreational level. Before participation, all individuals were thoroughly informed about the risks and advantages of all study procedures and provided written informed consent to participate. All procedures were approved by the ethical committee of Exercise Science & Training of the Faculty of Human Sciences of the University of Würzburg (EV2024/4‐1509) and conducted in accordance with the Declaration of Helsinki (Harriss & Atkinson, 2009; World Medical Association, 2013).

### 2.2 Experimental design

**FIGURE 1:**
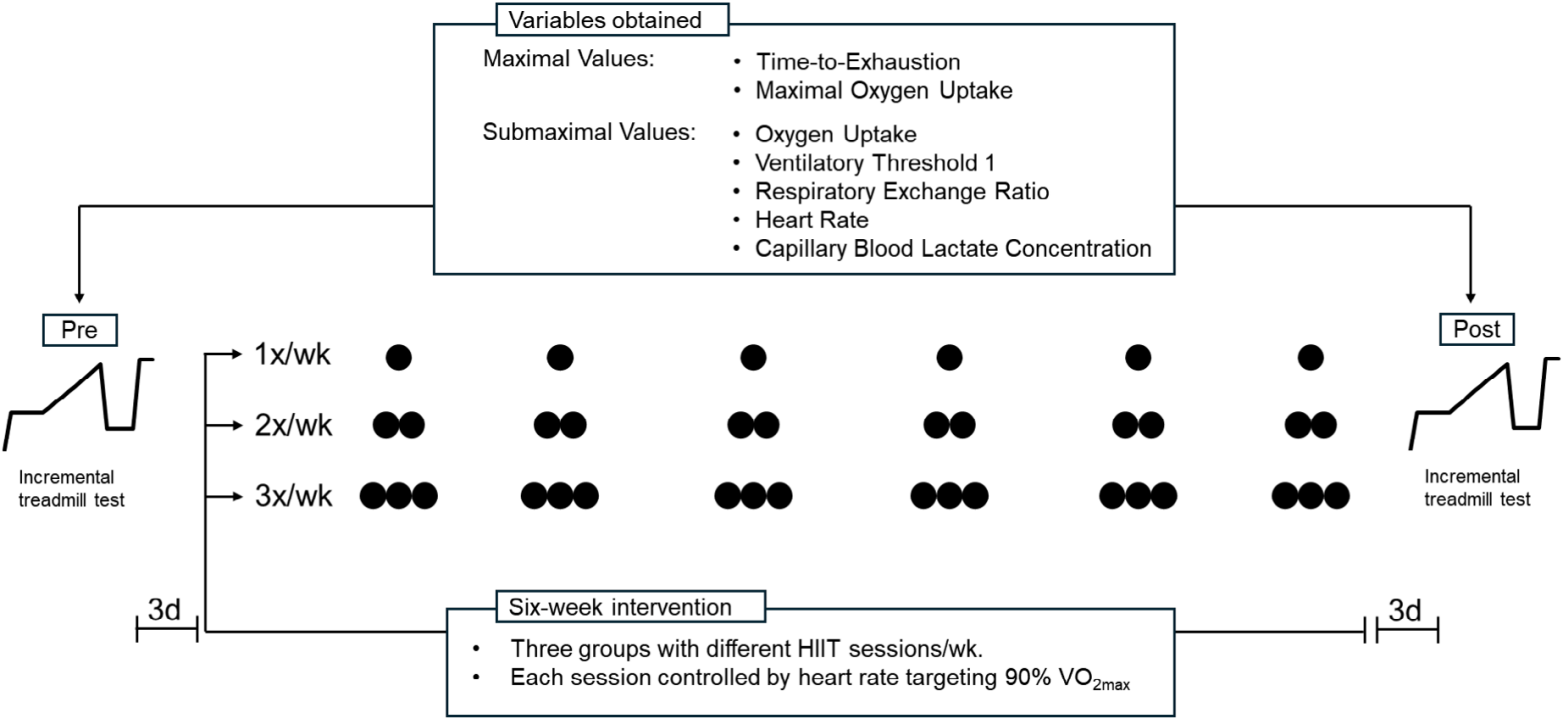
Study design and variables obtained during pre‐ and post‐testing.

Figure 1 illustrates the overall intervention design, including the parameters assessed.

Prior to the 6‐week training program, each participant visited the laboratory for baseline testing, which included cardiorespiratory, metabolic, and performance assessments in connection with incremental treadmill running. These tests were repeated after completing the 6‐week intervention.

A 6‐week training intervention was chosen based on previous findings demonstrating that this duration is sufficient to elicit meaningful performance and physiological adaptations in recreational runners (Zinner et al., 2018).

### 2.3 Testing procedures

All participants completed an incremental running test on a motorized treadmill (Mercury, h/p/cosmos sports and Medical GmbH, Nussdorf‐Traunstein, Germany). The test began with a 5‐minute steady‐state run at 7 km·h^−1^ for women and 8 km·h^−1^ for men, with a starting treadmill inclination of 1%. The incremental phase commenced at these speeds, increasing by 1 km·h^−1^ every minute until reaching 16 km·h^−1^ (women) or 17 km·h^−1^ (men). Subsequently, the treadmill incline increased by 1% per minute until participants reached voluntary exhaustion.

After a 3‐min active recovery period (3 km·h^−1^, 1% incline), a verification trial was conducted at 110% of maximal velocity to confirm VO_2max_ measurements. To ensure maximal effort, verbal encouragement was provided throughout. Blood lactate concentrations were measured via capillary blood samples collected from the earlobe before the test, immediately after steady‐state running, and post‐exhaustion using a Lactate Pro 2 analyzer (Arkray KDK).

Heart rate and pulmonary gas exchange were monitored continuously using a Polar Wear Link System (Polar Electro OY, Kempele, Finland) and a Cortex Metamax 3B gas analyzer (Biophysic GmbH, Leipzig, Germany), which were calibrated before each test using high‐precision gas and a 3‐L syringe. The variables assessed during the incremental test at maximum exertion included VO_2max_, maximum heart rate (HR_max_), and the time to exhaustion. Submaximal variables obtained at 7 (female) and 8 (male) km·h^−1^ included oxygen uptake (a surrogate variable for running economy), heart rate, respiratory exchange ratio (RER), and blood lactate concentration. Ventilatory threshold 1 (VT_1_) was calculated as described elsewhere (Esteve‐Lanao et al., 2005; Seiler & Kjerland, 2006; Zapico et al., 2007).

### 2.4 Training intervention

Participants were randomly assigned to one of three groups based on training frequency: one (1×·week^−1^), two (2×·week^−1^), or three (3×·week^−1^) 4 × 4‐min HIIT sessions per week. Training intensity was individualized using HR_max_ and maximum treadmill speed (V_max_) from baseline testing.

During the first two sessions, participants trained at 85%–90% of V_max_ and 85%–90% of HR_max_, based on values obtained from pre‐testing. These intensities were incrementally increased, corresponding to velocity increments of 0.2–0.5 km·h^−1^. By the final session, participants reached intensities of approximately 95% V_max_ and HR_max_. The active rest periods consisted of 3‐min bouts at 4 km·h^−1^. To ensure adherence to the prescribed training intensity, participants wore Polar H10 heart rate sensors (Polar Electro OY, Kempele, Finland) during all sessions.

### 2.5 Statistical analysis

All data were calculated with conventional procedures and presented as mean values and standard deviation. All data were checked for normality as well, with no data necessary for further transformation. A two‐way mixed design ANOVA was performed for each outcome variable, with time (pre vs. post) as the within‐subject factor and training frequency group (1×, 2×, 3× per week) as the between‐subject factor. This approach allowed for the examination of both main effects and interaction effects. Where a significant main effect of time was observed, Bonferroni‐corrected post hoc comparisons were conducted to identify within‐subject changes. Post hoc comparisons between groups were reported for descriptive purposes but interpreted with caution in the absence of significant interaction effects. Statistical significance was set at *p* < 0.05. Additionally, Cohen's d effect sizes and their 95% confidence intervals were calculated for pre‐post changes within groups, using thresholds of 0.20 (trivial), 0.50 (moderate), and 0.80 (large), to complement significance testing and aid interpretation considering the exploratory study (Cohen, 1988). This study was exploratory in nature, and no a priori power calculation was performed due to the practical limitations of recruiting large samples for a supervised intervention. Given the relatively small sample size and associated risk of type II error, effect sizes with confidence intervals are reported to provide a more comprehensive representation of the data beyond *p*‐values, acknowledging that the analyses are exploratory rather than confirmatory.

## 3 RESULTS

Out of the targeted 6, 12, and 18 sessions for the 1xHIIT, 2xHIIT, and 3xHIIT groups, the participants achieved adherence rates of 100%, 96%, and 91%, respectively.

The mean data for all training groups, along with the results of the statistical analysis, is presented in Table 1. Effect size calculations revealed moderate to large effects (i.e., *d* > 0.2) for pre‐ to post‐intervention changes in VO_2max_, TTE, and submaximal heart rate in participants training two or three times per week. Additionally, moderate effect sizes were observed for submaximal oxygen uptake, submaximal heart rate, and submaximal capillary blood lactate concentration.

**TABLE 1:** Mean data, standard deviation (SD), and 95% confidence intervals for all training groups and results of the statistical analysis.

	Parameter	Frequency (1 week^−1^)	Pre	Post	Paired *t*‐test	Effect size		ANOVA	*p* (pre/post × group)
			Mean ± SD	Mean ± SD	*p* (pre/post)	*d*	95% CI (d)	*p* (pre/post)	
Maximum	Oxygen uptake (mL·kg^−1^·min^−1^)	1	53.0 ± 7.0	53.6 ± 6.7	0.60	0.09	−0.68 to 0.86	0.004	0.167
		2	53.5 ± 8.1	57.6 ± 9.2	0.02	0.48	−0.28 to 1.24		
		3	48.5 ± 5.1	51.2 ± 3.6	0.01	0.61	−0.41 to 1.63		
	Time‐to‐exhaustion (s)	1	598 ± 115	601 ± 132	0.92	0.02	−0.77 to 0.77	0.002	0.080
		2	601 ± 136	667 ± 133	0.00	0.49	−0.27 to 1.25		
		3	575 ± 72	626 ± 59	0.02	0.77	−0.30 to 1.84		
Submaximum	Oxygen uptake (mL·kg^−1^·min^−1^)	1	29.1 ± 1.7	28.1 ± 2.1	0.24	−0.49	−1.31 to 0.33	0.751	0.237
		2	30.5 ± 4.3	31.0 ± 4.8	0.40	−0.11	−0.83 to 0.61		
		3	25.9 ± 4.4	26.0 ± 3.8	0.83	−0.02	−0.94 to 0.90		
	Ventilatory threshold 1 (L·min^−1^)	1	1.62 ± 0.54	1.57 ± 0.52	0.40	−0.09	−0.86 to 0.68	0.598	0.376
		2	2.10 ± 0.55	2.02 ± 0.46	0.36	−0.16	−0.88 to 0.56		
		3	1.37 ± 0.26	1.43 ± 0.24	0.16	0.24	−0.70 to 1.18		
	Respiratory exchange ratio (a.u.)	1	0.86 ± 0.04	0.84 ± 0.05	0.20	−0.44	−1.25 to 0.37	0.285	0.694
		2	0.87 ± 0.03	0.87 ± 0.03	0.45	0.00	−0.72 to 0.72		
		3	0.84 ± 0.03	0.84 ± 0.02	0.98	0.00	−0.92 to 0.92		
	Heart rate (1 min^−1^)	1	139 ± 17	135 ± 15	0.29	−0.25	−1.03 to 0.53	0.099	0.848
		2	145 ± 24	139 ± 15	0.50	−0.30	−1.03 to 0.43		
		3	144 ± 20	140 ± 18	0.35	−0.21	−1.15 to 0.73		
	Capillary blood lactate concentration (mmol L^−1^)	1	1.90 ± 0.36	2.21 ± 0.87	0.32	0.47	−0.35 to 1.29	0.852	0.218
		2	2.47 ± 0.60	2.24 ± 0.72	0.21	−0.35	−1.09 to 0.39		
		3	1.60 ± 0.41	1.60 ± 0.40	1.00	0.00	−0.92 to 0.92		

Overall, the most favorable pre‐ to post‐intervention effect sizes were observed in participants completing 2 or 3 HIIT sessions per week, suggesting that these frequencies provide a more robust stimulus for improving endurance‐related physiological variables.

**FIGURE 2:**
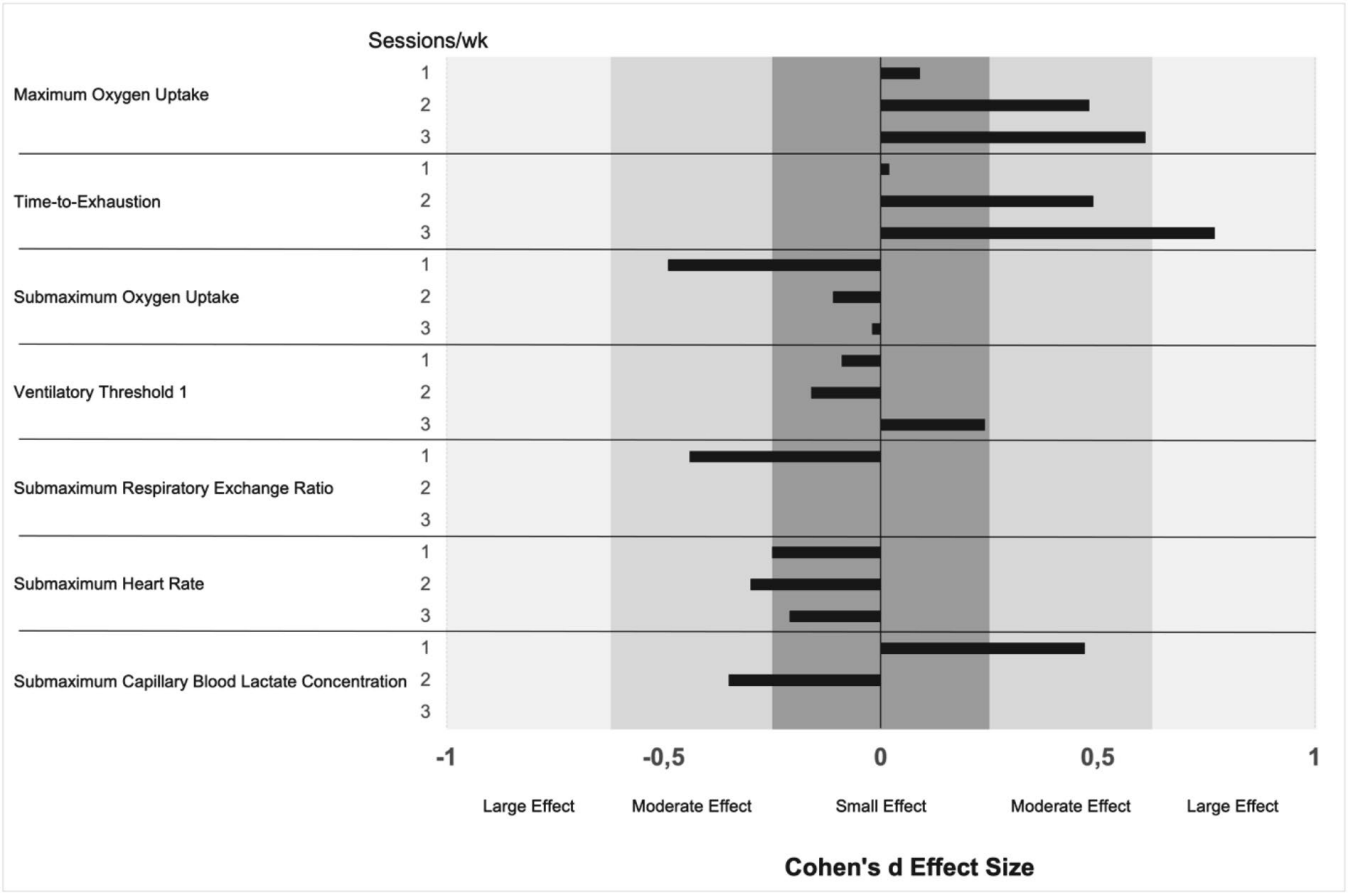
Cohen's d effect size calculation for each variable and amount of HIIT session per week.

ANOVA revealed statistically significant main effects of time for VO_2max_ (*d* = 0.09–0.61) and TTE (*d* = 0.02–0.77) across all groups (*p* < 0.05), indicating overall improvements from pre to post. The corresponding 95% confidence intervals for VO_2max_ ranged from [−0.68, 0.86] in the 1×·week^−1^ group to [−0.41, 1.63] in the 3×·week^−1^ group; for TTE, from [−0.77, 0.77] in the 1×·week^−1^ group to [−0.30, 1.84] in the 3×·week^−1^ group. All intervals included zero, indicating statistical uncertainty. Interaction effects (time × group) were not statistically significant, suggesting that changes from pre‐ to post‐intervention did not differ significantly between training frequencies. Group‐specific changes are therefore presented descriptively via effect sizes. The effect sizes for each training group are illustrated in Figure 2.

## 4 DISCUSSION

This exploratory study evaluated the effects of varying frequencies (once, twice, or thrice weekly) of 4 × 4‐min HIIT on key endurance variables in recreationally active adults. While significant improvements in VO_2max_ and TTE were observed across all training groups, no differences were found between the frequencies. These findings suggest that both 2 and 3 sessions per week provide a similar stimulus for cardiorespiratory improvements, with diminishing returns for increasing frequency beyond twice weekly.

The adherence rates in the present investigation were commendably high (91%–100%), suggesting the feasibility of HIIT across varying frequencies. However, the slightly lower adherence in the 3xHIIT group could reflect the practical challenges of sustaining higher training frequencies, even among motivated recreational runners. This observation supports the notion that twice‐weekly HIIT may strike an optimal balance between training load, recovery, and adherence, aligning with prior research highlighting diminishing returns with excessive frequency.

The improvement in VO_2max_ aligns with established research emphasizing the efficacy of HIIT in enhancing aerobic capacity through central (e.g., increased stroke volume) and peripheral (e.g., mitochondrial biogenesis) adaptations (Gibala & Jones, 2013). Although effect sizes were more pronounced in participants training two to three times weekly, the lack of significant interaction effects and the wide confidence intervals indicate limited precision of the estimates. Thus, the observed effect sizes should be regarded as descriptive and hypothesis‐generating rather than confirmatory.

Improvements in TTE across all groups underline the effectiveness of 4 × 4‐min HIIT in extending endurance performance. This enhancement likely reflects a combination of improved oxygen delivery and increased energy efficiency during sustained high‐intensity efforts. Although VT_1_ was assessed as an indicator of aerobic efficiency, no significant changes were observed across the groups. Thus, while TTE improved notably in the 2× and 3× per week groups, these gains cannot be directly attributed to measurable changes in ventilatory threshold. The absence of significant changes in running economy further suggests that the intervention period may have been insufficient to induce measurable adaptations in biomechanical or neuromuscular efficiency, which typically require prolonged or concurrent training modalities (Prieto‐Gonzalez & Sedlacek, 2022). Moreover, as the training was conducted at higher intensities than the submaximal speeds used for assessment, participants may not have spent sufficient time practicing the specific movement patterns at these lower velocities, limiting motor learning effects and potential improvements in exercise economy.

Participants training twice or thrice weekly showed a trend toward reduced submaximal heart rate (HR_submax_), which aligns with previous findings suggesting that intensified endurance training may decrease submaximal heart rate at a given running velocity, reflecting improved cardiovascular efficiency (Andrew et al., 1966; Clausen et al., 1970; Ekblom et al., 1968). Although these changes did not reach statistical significance, such trends could be linked to increased stroke volume and vagal tone, further supporting the role of HIIT in enhancing submaximal cardiovascular responses. However, changes in ventilatory thresholds and submaximal capillary blood lactate concentrations were trivial to moderate and varied across groups, indicating the nuanced and individualized nature of these adaptations. Nevertheless, the lack of statistical robustness and the overlap of the 95% confidence intervals with zero suggest limited precision of these estimates, and they should therefore be interpreted cautiously.

The variability in individual responses warrants attention (Zinner et al., 2023). While the mean effect sizes indicated moderate‐to‐large improvements, inter‐individual differences were apparent, with some participants exhibiting limited or no response to specific frequencies. Such variability emphasizes the need for personalized training approaches that account for individual recovery, baseline fitness, and responsiveness to different training stimuli. Future research should explore whether extended intervention durations or hybrid training modalities (e.g., combining HIIT with low‐intensity steady‐state exercise) could mitigate non‐responsiveness.

### 4.1 Strengths and limitations

This study has several strengths, including a well‐structured design with high adherence rates (91%–100%), individualized training intensity, and robust assessments of key endurance variables. Furthermore, its exploratory design constitutes a strength in itself, as it allows for the generation of preliminary evidence under ecologically valid conditions, the identification of meaningful trends despite limited statistical power, and the formulation of hypotheses to be addressed in future confirmatory research. However, limitations must also be acknowledged. The relatively small sample size may have reduced statistical power, increasing the risk of type II error and potentially obscuring subtle between‐group differences. No a priori power calculation was performed, as the study was designed to be exploratory and constrained by the practical challenges of recruiting larger samples for a supervised intervention. Although effect sizes and their 95% confidence intervals were reported to provide a more comprehensive characterization of the data, all confidence intervals included zero, underlining the limited precision of the estimates and restricting the strength of the conclusions. In addition, the six‐week duration, while sufficient for aerobic improvements, may have been too short to detect adaptations in submaximal variables such as running economy or neuromuscular function. Variations in participants' background activity and the absence of direct strength assessments further restrict the interpretation of the results. Future research should address these issues with larger, adequately powered samples, longer intervention periods, and more comprehensive assessments, while also exploring individual variability in responsiveness to different training frequencies.

## 5 CONCLUSION

In conclusion, this exploratory study suggests that 2–3 weekly sessions of 4 × 4‐min HIIT may be effective for improving VO_2max_ and TTE in recreationally active individuals, with no clear additional benefit from increasing the frequency to three sessions per week. These preliminary findings support the potential value of twice‐weekly HIIT as a practical and time‐efficient strategy for enhancing key endurance parameters, while acknowledging the limited precision of the estimates. Future studies with larger, adequately powered samples and longer intervention periods should confirm these trends, integrate dietary monitoring, and further explore mechanisms underlying inter‐individual variability to optimize training recommendations.

## AUTHOR CONTRIBUTIONS

Billy Sperlich, Mascha Lenk conceptualized the study. Mascha Lenk, Manuel Matzka, Lukas Lauber, Philipp Kunz performed the experiments and data analysis. Mascha Lenk, Billy Sperlich drafted the manuscript; Manuel Matzka, Philipp Kunz, Lukas Lauber revised the manuscript. All authors have read and approved the final manuscript.

## FUNDING INFORMATION

This research did not receive any specific grant from funding agencies in the public, commercial, or not‐for‐profit sectors.

## CONFLICT OF INTEREST STATEMENT

The authors declare that they have no competing interests.

## ETHICS STATEMENT

All procedures were approved by the ethical committee of Exercise Science and Training of the Faculty of Human Sciences of the University of Würzburg (EV2024/4‐1509) and conducted in accordance with the Declaration of Helsinki.

## CONSENT

All participants signed written informed consent to participate.

## Data Availability

All data are available from the corresponding author on request.
